# First Evidence of the Potential of Postharvest Hesperidin Treatments: Effects on Strawberry Quality During Storage

**DOI:** 10.3390/foods14162837

**Published:** 2025-08-16

**Authors:** Mihaela Iasmina Madalina Ilea, Huertas María Díaz-Mula, Christian Fernández-Picazo, Pedro Javier Zapata, Alicia Dobón-Suárez, Salvador Castillo, Fabián Guillén

**Affiliations:** Postharvest Research Group of Fruit and Vegetables, Institute for Agrifood and Agri-Environmental Research and Innovation (CIAGRO-UMH), Miguel Hernández University of Elche, Ctra. Beniel km. 3.2, 03312 Orihuela, Spain; mihaela.ilea@goumh.umh.es (M.I.M.I.); christian.fernandez01@goumh.umh.es (C.F.-P.); adobon@umh.es (A.D.-S.); scastillo@umh.es (S.C.)

**Keywords:** citrus flavonoids, *Fragaria x ananassa*, cold storage, oxidative stress, bioactive compounds

## Abstract

Strawberries (*Fragaria x ananassa* Duch.) are highly perishable fruits that rapidly lose their quality properties, even when stored under cold conditions. The purpose of this research was to study the effectiveness of hesperidin (10, 50, and 100 mg L^−1^) to preserve harvest-ripe strawberry quality during cold storage (2 °C). The data obtained indicate that hesperidin treatments were able to delay fruit metabolism and thus weight loss, while maintaining firmness and delaying colour evolution, obtaining positive results even with the lower concentrations applied. Strawberries treated with hesperidin exhibited a cell membrane with greater integrity, as reflected by a lower loss of electrolytes, resulting from reduced oxidation degradation. In addition, these strawberries maintained a higher concentration of chlorophylls in the calyx during storage, which could be due to a better antioxidant balance and a more effective preservation of their qualities. In this regard, the levels of bioactive substances, including total phenolics and the major anthocyanin compounds present in strawberries, were delayed in hesperidin-treated strawberries. This is the first report highlighting the effectiveness of hesperidin as a postharvest treatment in fruit, specifically in strawberries, delaying senescence. These results suggest that hesperidin, either by itself or in hesperidin-rich extracts, could become a valuable tool for postharvest fruit preservation.

## 1. Introduction

Strawberries (*Fragaria x ananassa*) are a non-climacteric and very perishable fruit, with a short shelf life, even during cold storage. Its high respiration rate and high susceptibility to mechanical damage and microbial incidence deteriorate strawberry quality rapidly [1]. Different strategies have been studied to maintain the quality properties of this fruit. Among these, physical treatments such as low temperatures, thermal treatments, active and modified atmosphere packaging, or light treatments have demonstrated positive effects [2,3,4,5,6]. Other technologies involving individual strawberry freezing, applications of high pressures, or short hot treatments have also been evaluated [7]. Additionally, several chemical treatments have been applied, delaying fruit senescence in strawberries, including 1-MCP [8], plant elicitors such as γ-aminobutyric acid (GABA), melatonin, phenylalanine or methyl jasmonate [1,2,9,10]. One of the most studied technologies in strawberries is the use of edible coating formulations [11,12,13] based on natural extracts [2], including the application of citrus essential oils as edible coatings or as components of active packaging [14,15]. All these strategies have also been applied alone or in combination to enhance the individual effects of the different technologies [1,2]. Some of them have shown interesting effects, maintaining bioactive compound content, including anthocyanins and other secondary metabolites during storage [16,17].

Recently, the use of polyphenolic extracts obtained from plants, as well as the use of pure polyphenols, such as chlorogenic acid, has demonstrated their contribution to postharvest preservation in fruits, primarily due to their antioxidant activity. In this regard, postharvest treatments in different fruit species based on polyphenolic extracts have shown benefits in delaying senescence and reducing quality losses during storage, as apple or tea extracts rich in these bioactive compounds [16,18,19].

Hesperidin is a phenolic compound, particularly a flavonoid, commonly present at elevated levels in citrus species, including oranges, lemons, and mandarins [20]. Several studies have shown that hesperidin can provide health benefits, including antitumoral effects in various organs, such as the colon [21], bones [22], or human skin [23], as well as cardioprotective effects against chemotherapy side effects [24]. Additionally, it has been demonstrated that hesperidin has a positive impact on improving cognitive status, reducing depression in animal models [25], as well as its antimicrobial, antiviral, and hepatoprotective actions. These effects are primarily based on maintaining the antioxidant status, which prevents the oxidative imbalance that leads to more than 100 illnesses in various animal models [20].

Although hesperidin is one of the most abundant flavonoids in citrus fruits, to date, no study has specifically evaluated the postharvest impact of hesperidin or hesperidin-rich extracts on fruit quality. Nevertheless, although citrus peel extracts rich in bioactive compounds have been studied, they have only been applied as additives in edible coatings and never assessed as isolated treatments in several fruit species such as grapes, apples, litchis, and tomatoes, showing beneficial effects in delaying senescence and preserving quality [26,27,28,29,30]. Despite their richness in phenolics, such as hesperidin, none of these studies have directly evaluated the hesperidin content or related the positive effects to this particular flavonoid.

Alternatively, essential oils extracted from different citrus species have been applied to control postharvest disorders; however, these oils do not contain hesperidin because they are traditionally obtained through the distillation process [31,32]. This process involves extracting volatile compounds, such as monoterpenes and aromatic hydrocarbons. In contrast, hesperidin is a non-volatile, thermolabile compound that degrades at high temperatures and must be obtained using solvent-based methods or alternative techniques [33]. Citrus peel extracts used in food preservation typically employ solvent extraction techniques, allowing for the presence of hesperidin in the resulting matrices [34]. For these reasons, this study presents the first investigation into the potential of hesperidin in any fruit species as a postharvest strategy to increase shelf life, making this study a novel approach to using this flavonoid as a technological tool in fruit preservation.

## 2. Materials and Methods

### 2.1. Fruit Material and Experimental Conditions

The strawberry (*Fragaria x ananassa* Duch.) cultivar used in this study (cv. Florida Radiance) was manually harvested with a red ripening stage (fully mature fruit) from a commercial plot in Huelva (Spain). That day, the fruits arrived in the laboratory via refrigerated transport, where 1020 strawberries, with no external damage or pathological symptoms, were classified according to uniformity in colour and size. The laboratory was maintained at a controlled temperature of 20 °C. After selection, strawberries were sanitized by immersion in a 10 ppm sodium hypochlorite solution (Merck (Darmstadt, Germany; Cat. No. 1.05614.2500; CAS No. 7681-52-9; 6–14%) for 3 min to minimize the influence of heterogeneous microbial load on the experimental results. Additionally, a batch of 60 strawberries was used to assess the initial quality parameters upon arrival at the laboratory (day 0). Then, all the fruits were divided into 3 replicates of 20 strawberries for each treatment and sampling day. Hesperidin (Tokyo Chemical Industry, Tokyo, Japan; Cat. No. H0049; CAS No. 520-26-3; purity > 90%) was previously dissolved to reach each concentration with 1 mL of ethanol. A similar volume of ethanol (Merck, Darmstadt, Germany; Cat. No. 1.11727.2500; CAS No. 64-17-5 purity ≥ 99.9%) was added to the control solution. For hesperidin treatments, immersions in 10, 50, and 100 mg L^−1^ solutions were applied for 10 min, and distilled water immersions served as the control treatment for 10 min as well. All hesperidin and control solutions contained Tween 20 (Sigma-Aldrich, Madrid, Spain) 0.05% *v*/*v*. After treatment, the fruits were left to dry at 20 °C for 30 min and then stored at 2 °C and 90% RH for 12 days for subsequent shelf life determinations.

### 2.2. Postharvest Quality Traits

Fruit weight loss was evaluated per replicate at the beginning of the experiment and during storage and expressed as a percentage of the initial weight (*n* = 3). Fruit firmness was determined using a 5 mm diameter Magness-Taylor probe (Stable Micro Systems, Godalming, UK), which penetrated the pulp to a depth of 5 mm at a constant speed of 1.0 mm s^−1^. The maximum force required for penetration, measured in Newtons (N), was recorded for each strawberry individually.

Membrane integrity was evaluated through electrolyte leakage (EL) analysis and malondialdehyde (MDA) content following Ilea et al. [1] recommendations with some modifications. For each replicate, 20 cylindrical tissue sections (3 mm diameter and 3 mm thickness) were excised from equatorial strawberry sections, specifically from fully red-coloured tissue (avoiding the white central area). Cylinders were rinsed and subjected to triple washing (3 min per wash) in deionized water (Millipore Corp., Darmstadt, Germany) to remove surface electrolytes. Subsequently, the tissue cylinders The samples were placed in 50 mL of deionized water and kept at room temperature with constant agitation for 1 h. Subsequently, the initial conductivity (C_1_) was recorded at 20 °C. To achieve complete EL, the samples were then heated at 100 °C for 15 min and then tempered at room temperature to evaluate the final conductivity (C_2_). EL was expressed as the percentage ratio of C_1_ to C_2_ (C_1_/C_2_ × 100) (*n* = 3). To quantify MDA content, 2.5 g of pulp was homogenized with 10 mL of cold 1% (*w*/*v*) trichloroacetic acid (TCA) (Ultraturrax, T18 basic, IKA, Berlin, Germany) for 1 min. The mixture was then centrifuged at 10,000 rpm for 10 min at 4 °C. A 1 mL aliquot of the resulting supernatant was diluted with 1 mL of TCA. Then, 6 mL of 0.67% (*w*/*v*) thiobarbituric acid (TBA) solution was added, and the mixture was incubated in a water bath at 95 °C for 30 min, followed by immediate cooling in an ice bath to stop the reaction. Absorbance readings were obtained at 450, 532, and 600 nm using a UV-Vis spectrophotometer (Model 1900, Shimadzu, Kyoto, Japan). Results were expressed in µmol MDA per kg of fresh weight. Each analysis was conducted in duplicate for each replicate (*n* = 3).

To evaluate respiration and ethylene production, 10 strawberries per treatment and replicate were selected randomly and deposited into small glass containers (0.5 L), allowing them to accumulate headspace gases for 60 min at room temperature. After this period, 1 mL gas samples were obtained in sextuplicate per replicate through a septum using 1 mL syringes. Following the conditions described by Martínez-Romero et al. [35], ethylene (nmol kg^−1^ h^−1^) and carbon dioxide concentrations (mg CO_2_ kg^−1^ h^−1^) were quantified with 3 syringes for each parameter using a gas chromatograph (GC-2010 for ethylene and GC-14B for CO_2_; Shimadzu Europa GmbH, Duisburg, Germany).

The evaluation of the total soluble solids (TSS) and the titratable acidity (TA) in each replicate involved longitudinally sectioning and then transversely sectioning the strawberries at the equator. The apical half of each fruit (characterized by more uniform colour) was selected for analysis. Twenty sub-samples per replicate were obtained and manually pressed through a double layer of cotton cloth to extract a homogenized and clarified juice. With this juice, TSS was evaluated using a digital refractometer (Atago PR-101, Atago Co., Ltd., Tokyo, Japan) at 20 °C, with results expressed as g 100 g^−1^ of fresh weight. TA was determined using 1 mL of juice diluted to 25 mL with distilled water, titrated with an automatic titrator (785 DMP Titrino, Metrohm, Herisau, Switzerland), and expressed as g of citric acid equivalents per 100 g of fresh weight.

Colour parameters were evaluated on the external surface area at three different points along the equatorial region for each strawberry with a colorimeter (CRC400, Minolta Camera Co., Tokyo, Japan). For this evaluation, the CIELAB system (L*, a*, b*) was used to express lightness (CIE L*) and chroma (CIE C*) and hue angle (CIE Hue*) values.

### 2.3. Total Chlorophylls and Polyphenolic Content

The total chlorophyll content (TCC) in strawberry calyces was taken from 20 strawberries per replicate (*n* = 3). Strawberry calyces were collected and pooled for each replicate to obtain 1 g of fresh tissue in duplicate. The sample was homogenized (Ultraturrax, T18 basic, IKA, Berlin, Germany) and extracted with 20 mL of 99.9% methanol for 1 min. The mixture was centrifuged at 10,000 rpm for 10 min at 4 °C to separate the supernatant. Absorbance readings were recorded at 665.2 nm and 652.4 nm using a UV-Vis spectrophotometer (Model 1900, Shimadzu, Kyoto, Japan). TCC was calculated according to the equations provided by Vu et al. [36] and expressed as mg g^−1^ of dry weight.

Total phenolic content (TPC) was evaluated from one longitudinal half of each strawberry, which was immediately frozen in liquid nitrogen and finely ground to a uniform powder. For each replicate, 5 g of frozen tissue was homogenized (Ultraturrax, T18 basic, IKA, Berlin, Germany) with 10 mL of a methanol: water solution (80:20, *v*/*v*) containing 2 mM sodium fluoride to inhibit polyphenol oxidase activity. The suspension was homogenized for 1 min and then centrifuged at 10,000× *g* for 15 min at 4 °C. The supernatant was collected and analyzed in duplicate per replicate using the Folin–Ciocalteu colorimetric method, following the protocol explained by Lezoul et al. [37] to evaluate several plant parts, including fruit. Then, a standard calibration curve (gallic acid) was prepared to quantify the samples, and the results were recorded as mg of gallic acid equivalents (GAE) per 100 g of fresh sample.

For individual anthocyanin quantification, using liquid nitrogen, strawberries were pulverized for each replicate, and then 5 g were immediately extracted with 5 mL of methanol acidified with 1% HCl (*v*/*v*), homogenizing for 1 min (Ultraturrax, T18 basic, IKA, Berlin, Germany). The homogenate was filtered through a 0.45 µm filter to obtain a clear extract. From each replicate, three dilution levels (undiluted, 1:5, and 1:10, *v*/*v*) were prepared before chromatographic analysis. Quantification and identification of anthocyanins were carried out using a Shimadzu LC–MS/MS 8050 triple quadrupole mass spectrometer coupled with an HPLC system (Shimadzu, Kyoto, Japan) equipped with a photodiode array detector (SPD-M40). Separation was achieved on a Mediterranea SEA18 column (100 mm × 2.1 mm, 2.2 µm; Teknokroma, Barcelona, Spain) held at 50 °C. The mobile phase was composed of solvent A (water with 0.1% formic acid, *v*/*v*) and solvent B (acetonitrile with 0.1% formic acid, *v*/*v*). Elution was conducted at a flow rate of 0.4 mL min^−1^ under the following gradient: isocratic 99% A for 1 min; linear increase of B from 1% to 40% between 1–15 min; ramp to 100% B from 15–23 min; return to 99% A in 2 min, followed by a 5-min isocratic phase and column re-equilibration. A 10 µL volume was injected for analysis. UV–Vis spectra were collected across a wavelength range of 200–600 nm. Anthocyanin peaks were identified based on their retention times and UV–visible profiles, which were compared to external standards for the quantification of the different anthocyanins (mg 100 g^−^^1^). The system was operated, and data were processed using LabSolutions LC-MS software (version 5.98, Shimadzu). Each analysis was carried out in triplicate.

### 2.4. Statistical Analysis

The data were presented as the mean ± standard error (SE) of three replicates (*n* = 3) of 20 strawberries and were subjected to analysis of variance (ANOVA). When significant differences were detected (*p* < 0.05), Tukey’s honest significant difference (HSD) test was applied to compare group means, and results were recorded with different lowercase letters within each sampling date. All statistical determinations were conducted with the SPSS software (version 22.0; IBM Corp., Armonk, NY, USA).

## 3. Results and Discussion

### 3.1. Effect of Hesperidin Treatments on Weight Loss, Fruit Firmness, MDA, and EL

**Figure 1:**
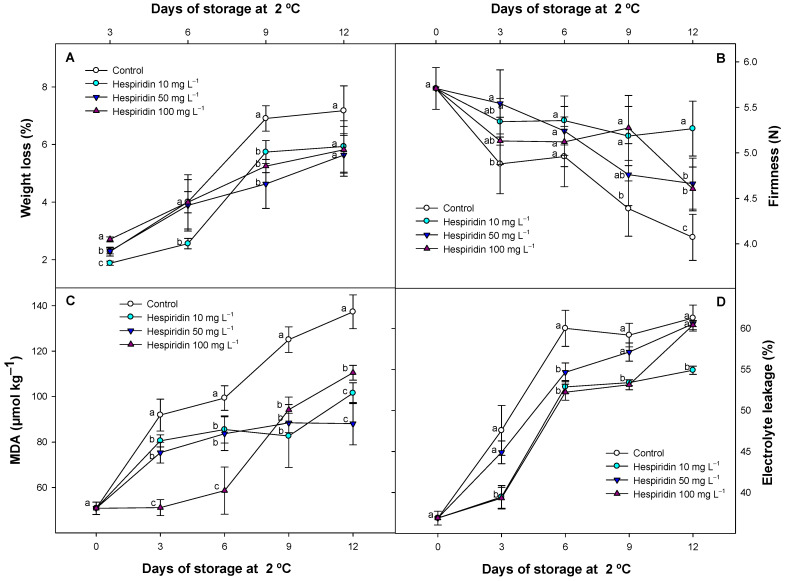
Changes in weight loss (%, (**A**)), firmness (Newtons, (**B**)), Malondialdehyde (μmol kg^−1^, (**C**)), and electrolyte leakage (%, (**D**)), in untreated strawberries and those exposed to several hesperidin concentrations throughout storage at 2 °C. Data represent the mean ± SE (*n* = 3). Distinct lowercase letters among each day of sampling are significantly different (*p* < 0.05) between treatments.

The applications with hesperidin at different concentrations displayed a significant (*p* < 0.05) effect on the weight loss evolution throughout the cold storage of strawberries (Figure 1A). Hesperidin treatments effectively delayed this parameter compared to control batches. After 9 days of storage, while control fruits exhibited a 6.90 ± 0.44% weight loss, hesperidin applications resulted in 5.73 ± 0.40%, 4.63 ± 0.85%, and 5.25 ± 0.23% weight loss at 10, 50, and 100 mg L^−1^, respectively.

A similar trend was observed for fruit firmness during storage, as hesperidin concentrations delayed this parameter compared to control batches along this experiment (Figure 1B). Even the lowest hesperidin concentration showed a significant effect, delaying the loss of fruit firmness, maintaining similar values at the end of the study (5.26 ± 0.30 N) to those found at day 0. However, the control fruit firmness fell to 4.07 ± 0.25 N after 12 days of storage. To evaluate the probable connection of these two parameters with membrane integrity, MDA and EL were assessed (Figure 1C,D). Results showed that all the hesperidin concentrations successfully delayed the MDA content and the membrane permeability. In both cases, no dose-dependent effect was observed, although at the beginning of the study, the highest hesperidin concentration was the most effective in delaying MDA accumulation. However, the intermediate concentration (50 mg L^−1^) displayed the lowest MDA accumulation, and the control fruit the highest MDA content (88.05 ± 9.27 and 137.33 ± 7.44 µmol kg^−1^, respectively). Regarding this parameter, the control batches also exhibited the highest permeability during storage compared to the hesperidin batches (Figure 1D). At the end of the study, the only hesperidin treatment that maintained significant (*p* < 0.05) differences compared to the other batches was the lowest concentration (10 mg L^−1^), which delayed membrane degradation by 5% compared to the control fruit and the different treatments applied.

During storage, hesperidin treatments maintained strawberry quality by delaying various parameters associated with structural deterioration in plant tissues. Regarding weight loss, hesperidin treatments significantly delayed this parameter at all concentrations assayed, suggesting effective control of the transpiration process and a delay in respiratory metabolism. In general, fruit weight loss increases during storage due to the breakdown of their cell structure, which enhances transpiration [38]. Cell breakdown is directly related to fruit softening; however, hesperidin treatments delayed fruit firmness loss because weight loss and fruit softening are interrelated. A higher water loss leads to a decrease in cellular turgidity, thereby increasing fruit softening [39]. Other biomolecules and polyphenols with antioxidant activity, such as GABA or chlorogenic acid, have demonstrated the ability to reduce weight loss, maintain fruit firmness by reducing membrane cell oxidation, and preserve turgidity in strawberries and other fruit species [1,18]. For this reason, the delay in these parameters is likely mainly due to the antioxidant protection provided by postharvest treatments based on hesperidin, another antioxidant biomolecule. In fact, at a cellular level, MDA evaluated in these strawberries was significantly reduced with hesperidin treatments. As MDA is a direct marker of cell membrane oxidation in plant tissues, these results indicate that hesperidin contributes to maintaining strawberry quality through mechanisms related to membrane maintenance, thereby reducing oxidative stress. A lower EL in hesperidin-treated fruit supported these effects in this study, even with the lower concentrations applied. Although there is no information regarding hesperidin treatments in fruit species, several studies have found that polyphenol extracts and pure polyphenols, applied in solutions or as edible coatings, can delay these structure-related parameters by maintaining membrane integrity and reducing oxidative stress [2,18,40,41,42]. In fact, in strawberries and other fruit species, it has been observed that an increase in cellular antioxidant activity (particularly that mediated by antioxidant enzymes such as superoxide dismutase (SOD), catalase (CAT), peroxidase (POD), and ascorbate peroxidase (APX)) contributes to delaying membrane degradation. This effect is related to a lower lipid peroxidation, reduced water loss, and the maintenance of fruit firmness during storage [17,40]. Several studies using antioxidant bioactive compounds have demonstrated improved antioxidant enzyme activity, reduced malondialdehyde accumulation, and better preservation of structural integrity and quality traits under cold storage conditions [10,17].

### 3.2. Effect of Hesperidin Treatments on Respiration and Ethylene Production

**Figure 2:**
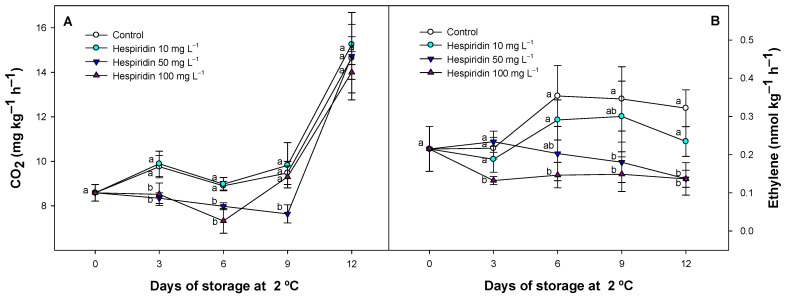
Respiration (mg CO_2_ kg^−1^ h^−1^, (**A**)) and ethylene production (nmoL kg^−1^ h^−1^, (**B**)) for the control batch and hesperidin-treated fruit throughout the storage period at 2 °C. Values represent the mean ± SE (*n* = 3). Distinct lowercase letters within each sampling day are significantly different (*p* < 0.05) among treatment applications.

The metabolic activity of strawberries is very high, reducing shelf life at room temperature, affecting firmness, and making them highly susceptible to mechanical damage, which facilitates microbial infection. The respiration of strawberries in this study was reduced in hesperidin-treated strawberries as compared with untreated batches (Figure 2A). The higher concentrations assayed displayed significantly (*p* < 0.05) lower values than those observed for control fruit and the lowest hesperidin concentration applied (10 mg L^−1^). After 6 days of refrigerated storage, the average values observed for the different batches were 8.90, 8.97, 7.98, and 7.32 mg CO_2_ kg^−1^ h^−1^ (for the control, 10, 50, and 100 mg hesperidin L^−1^ treated batches, respectively), displaying a dose-dependent pattern that was observed throughout the study.

Another indicator of fruit metabolism is the production of ethylene. Although strawberries are described as non-climacteric fruit, this gaseous hormone can affect quality because it is involved in other fruit changes, such as fruit colour evolution [43]. This parameter displayed a similar pattern to that observed for fruit respiration, with a dose-dependent effect provided by hesperidin concentrations (Figure 2B). The lowest ethylene values were produced in the hesperidin batches, whereas, in general, the highest ethylene production was observed in the control batch throughout the experiment.

Although no previous studies have reported the effects of hesperidin or citrus-derived polyphenol-rich extracts on fruit respiration, it is known that certain phenolic compounds (such as chlorogenic acid) can modulate respiratory metabolism by stimulating the GABA shunt pathway, reducing CO_2_ production in fruits [44]. This metabolic route provides a more energy-efficient alternative to glycolysis and β-oxidation under stress conditions, such as cold storage, by enabling faster ATP generation with reduced oxygen consumption [45]. In our previous studies, we also observed this effect after applying chlorogenic acid to tomatoes [18]. It is therefore plausible that the reduced respiration rates observed in hesperidin-treated strawberries may be partially attributed to similar metabolic regulation, given the structural similarity of flavonoids and the antioxidant properties of hesperidin. In general, antioxidant bioactive compounds have been shown to reduce oxidative stress by enhancing the endogenous antioxidant defense system, which is crucial in mitigating reactive oxygen species (ROS), thereby reducing the cellular demand for energy [44,45]. Furthermore, the lower ethylene levels detected could also be linked to a reduced microbial load, particularly with *Botrytis cinerea*, a pathogen known to produce ethylene during infection. In this study, no visible fungal development was detected in any of the treatments (even for control batches). This may be due not only to the efficacy of refrigeration but also to the application of sodium hypochlorite as a sanitizing agent before storage, a method previously described as an effective preservation strategy for different fruit species, including strawberries [1,42]. The improved tissue integrity observed in hesperidin-treated batches in our previous results (Figure 1B or Figure 1D) could have further limited pathogen invasion, indirectly contributing to the reduction of ethylene biosynthesis.

### 3.3. Effect of Hesperidin Treatments on TSS and TA

**Figure 3:**
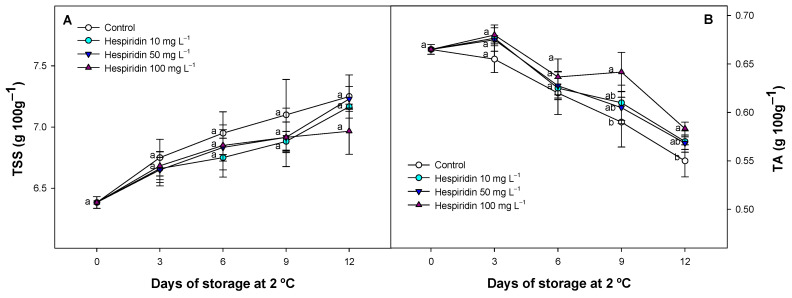
Evolution of total soluble solids (**A**) or titratable acidity (**B**) content, both represented as g 100 g^−1,^ in the control batches or hesperidin-treated applications throughout cold storage. Values are the mean ± SE (*n* = 3). Lowercase letters, which are different in each sampling period, are significantly different (*p* < 0.05) among treatments.

TSS and TA in strawberry samples increased and decreased as expected, respectively (Figure 3). However, the increased and decreased patterns were different depending on the treatment applied. In this regard, although non-significant (*p* > 0.05) differences were observed between the applied treatments in TSS content, average values of TSS accumulation were generally delayed in hesperidin-treated batches compared with the control fruit throughout the study (Figure 3A). Similarly, TA in hesperidin-treated batches displayed a significant (*p* < 0.05) delay in reduction during refrigerated storage, which was mainly observed with the highest hesperidin concentration after 9 days of storage, retaining almost 10% higher TA average values compared to the control fruit (Figure 3B).

In our study, TSS increased slightly during storage, which may be attributed to the concentration effect caused by water loss and a delayed pattern in these respiratory substrates. Similar increases have been reported in other cultivars where polyphenol and other bioactive compounds treatments lead to a TSS accumulation during cold storage [9,16]. However, the opposite trend has also been observed in other studies, particularly in strawberries harvested at different ripening stages, where TSS decreased over time due to sugar consumption through respiration, suggesting that physiological status can influence TSS evolution over time [1,46]. In our study, the delayed pattern in these respiratory substrates could be due to the lower metabolism of hesperidin-treated fruit, as observed in our previous data regarding the respiration process, where CO_2_ accumulation displayed lower values compared to control batches (Figure 2A). This relation has been described in general during postharvest fruit and vegetables previously [39] and specifically in polyphenol-treated fruit as extracts or as pure polyphenolic solutions on strawberry and other fruit species [18,41,47,48]. In the case of specific polyphenol applications, such as chlorogenic acid, the mechanism involved has been related to the stimulation of the GABA shunt pathway, in which the production of cell energy is more efficient with a lower number of enzymatic steps necessary to obtain ATP [44,45]. Thus, it is possible to maintain the energy requirements without increasing the catabolism of respiratory substrates in plant tissues. Additionally, it cannot be disregarded that a lower ROS content in strawberries and other fruits, provided by antioxidant compounds as postharvest treatment, maintains a better antioxidant balance, which affects the aging and integrity of plant tissues, thereby delaying the respiration process [46,49]. The effects of hesperidin treatment on TSS and TA may modify the sensory attributes of strawberries, potentially influencing the perceived sweetness and overall flavour. Therefore, future research should include formal sensory evaluations to confirm the organoleptic impact of the treatment and assess its consumer acceptability, which is crucial for potential commercial application.

### 3.4. Effect of Hesperidin Treatments on Strawberry Colour Evolution

**Figure 4:**
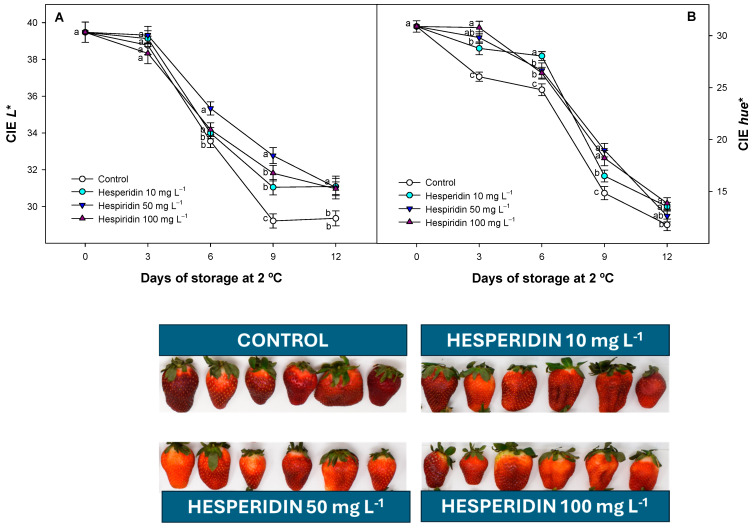
Effect of hesperidin treatment on different colour parameters (CIE L*, (**A**), and CIE hue*, (**B**)) evaluated in strawberries with several concentrations throughout cold storage. Lowercase letters that are different among treatments for each sampling date are significantly different (*p* < 0.05) for each period evaluated when the mean ± SE (*n* = 3) were compared. This figure includes the external appearance of strawberry fruits from each treatment, after 9 d of cold storage.

Colour parameters decreased during cold storage, but significant (*p* < 0.05) differences were observed between the different fruit batches evaluated (Figure 4A,B). CIE L* colour, which is related to fruit brightness, displayed a delayed pattern in hesperidin-treated batches compared to the control fruit (Figure 4A). Only minor differences were observed between hesperidin batches throughout the study, regardless of the applied concentration, and similar values were observed after 12 days of refrigerated storage. However, the CIE L* values of hesperidin-treated fruit were significantly higher (*p* < 0.05) than those of the control fruit after 6 days of storage, and this difference was maintained throughout the storage.

On the other hand, CIE hue* values, whose lower values are related to changes in darkening of the fruit surface affecting the colour tone, also displayed a decreased pattern for all the fruits tested (Figure 4B). However, the hesperidin-treated batches consistently displayed higher values throughout the entire experiment compared to the control batches. After 3 days of storage, significant (*p* < 0.05) differences were observed between all the treatments applied. In this regard, on day 3, the results observed were 26.05 ± 0.44 for the control batch, while the hesperidin-treated batches were 28.77 ± 0.61, 29.81 ± 0.55, and 30.78 ± 0.60 for the concentrations of 10, 50, and 100 mg L^−1^, respectively. These differences with the control fruit were maintained during the 12 days of storage.

Several studies have reported that colour degradation in fruits, including strawberries, is closely linked to moisture loss and the oxidative breakdown of pigments such as anthocyanins, which are the most abundant in this fruit [50]. A reduction in surface water content typically leads to darker fruit appearance, as reflected by declining CIE L* values, while oxidative changes in pigment compounds affect CIE hue* [1,50]. In previous studies, various polyphenolic treatments or polyphenolic extracts derived from polyphenol-rich sources, such as apples, pomegranate rinds, and tea leaves (excluding hesperidin or containing no hesperidin as a primary polyphenol) have been explored for their effectiveness in slowing down colour and pigment evolution during storage in different fruit species, including tomato, strawberries, and litchi [18,19,41,51]. These natural compounds, which include flavonoids and phenolic acids, contribute to the antioxidant protection of pigments during ripening and postharvest storage. For instance, the postharvest application of polyphenolic extracts has been associated with reduced water loss, thereby improving the retention of brightness and colour values in treated pitahaya [52]. Such effects confirm our observations in this study and those of previous studies on tomatoes [18], maintaining external quality. In nectarines, similar postharvest treatments with polyphenols, such as chlorogenic acid, have been shown to effectively detoxify ROS [53] and reduce PPO activity [41], supporting the role of polyphenols as functional and antioxidant agents for maintaining postharvest quality and external colour.

### 3.5. Effect of Hesperidin Treatments on TCC in Strawberry Calyces and TPC in the Fruit Flesh

The visual quality of strawberries is a key factor in consumer acceptance, with particular attention to the brightness of the fruit and the vivid green colour of the calyx. This visual appeal largely depends on two main pigment groups: chlorophylls in the calyx, which are associated with freshness and a just-harvested appearance, and reddish polyphenolic compounds such as anthocyanins in the fruit flesh, which contribute to the characteristic color of ripe strawberries. Degradation of these pigments during storage leads to discoloration and browning, negatively impacting the perception of quality [54].

**Figure 5:**
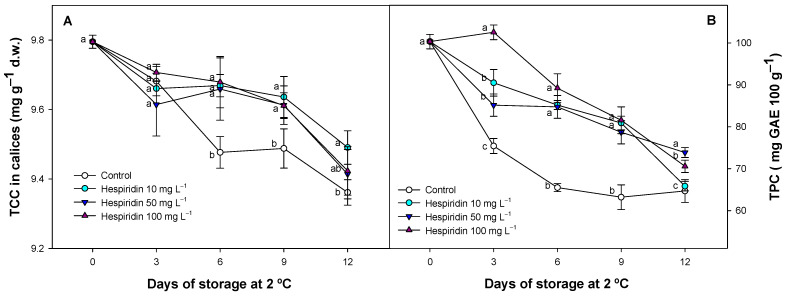
Chlorophyll evolution (TCC, mg g^−1^) (**A**) and polyphenolic pattern (TPC, described as mg of gallic acid equivalents 100 g^−1^) (**B**) for untreated strawberries and hesperidin-treated batches at several concentrations during 12 d at 2 °C. Values represent the mean ± SE (*n* = 3). Distinct lowercase letters for each evaluated period are significantly different (*p* < 0.05) between treatments.

The concentration of TCC and TPC decreased during storage in both calyces and fruit flesh, respectively (Figure 5).

In this study, hesperidin-treated strawberries showed a significant (*p* < 0.05) delay in the degradation of total chlorophyll content (TCC) in calyx tissues compared to control fruit (Figure 5A). Throughout the storage period, hesperidin treatments maintained higher chlorophyll levels, with no significant (*p* > 0.05) differences observed regardless of the applied hesperidin concentrations. However, these treatments generally showed higher values compared to the control batches during cold storage. Regarding TPC, the control fruit showed a rapid initial decrease, which was delayed in the hesperidin-treated batches. (Figure 5B). In contrast, hesperidin-treated fruit maintained a more stable phenolic content, with delayed degradation of these compounds. This was especially evident in the batch treated with 100 mg L^−1^, which generally maintained higher TPC values during storage. The absence of the typical early increase in TPC in control strawberries, often observed in less ripe strawberries prior to the later decline during storage [55], may be explained by the higher ripening stage of the fruit used in this study. In our previous work [1], where strawberries were harvested at approximately 70% red surface, TPC and anthocyanins still increased during early storage before decreasing [54], a pattern not observed here due to the more advanced maturity at harvest.

In this study, the present results support the role of hesperidin as an effective postharvest treatment for preserving pigment stability and antioxidant compounds, both of which are essential to maintaining the visual and nutritional quality of strawberries. The mechanism involved may be partially explained by the significantly lower ethylene production observed in hesperidin-treated fruit, as demonstrated in previous results (Figure 2B). Ethylene is a hormone known to regulate senescence processes, including the degradation of chlorophylls. In earlier studies [1,56,57] an inhibitor of ethylene production in strawberries (1-methylcyclopropene) reduced ethylene production, thus contributing to the delayed loss of green pigmentation in the calyx and polyphenols in the flesh, preserving cell wall integrity in strawberries. Additionally, the antioxidant capacity of hesperidin may have helped reinforce cellular redox homeostasis, thereby limiting the accumulation of ROS. This effect has been observed with antioxidant compounds as a postharvest treatment in calyces of different plant species [58,59]. The reduction in oxidative stress could help protect pigment-containing organelles such as chloroplasts and vacuoles, thereby preserving both chlorophylls in the calyx and anthocyanins in the fruit flesh. Previous studies have shown that polyphenolic extracts applied to strawberries [47,51], as well as individual polyphenols used as postharvest treatments in various fruit species [18,41], were effective in maintaining higher polyphenol levels, delaying colour degradation. This effect was associated with a lower oxidative status and enzymatic degradation in fruit tissues. In the present study, the lower MDA levels observed in hesperidin-treated strawberries (Figure 1C) can be attributed to an improved antioxidant status, which likely contributed to reduced oxidative damage during storage. As a result, hesperidin-treated strawberries could maintain a fresher appearance and more intense external colour throughout the storage period. However, to support this effect, individual anthocyanins have been evaluated in the following section.

### 3.6. Effect of Hesperidin Treatments on Individual Anthocyanins During Storage

**Figure 6:**
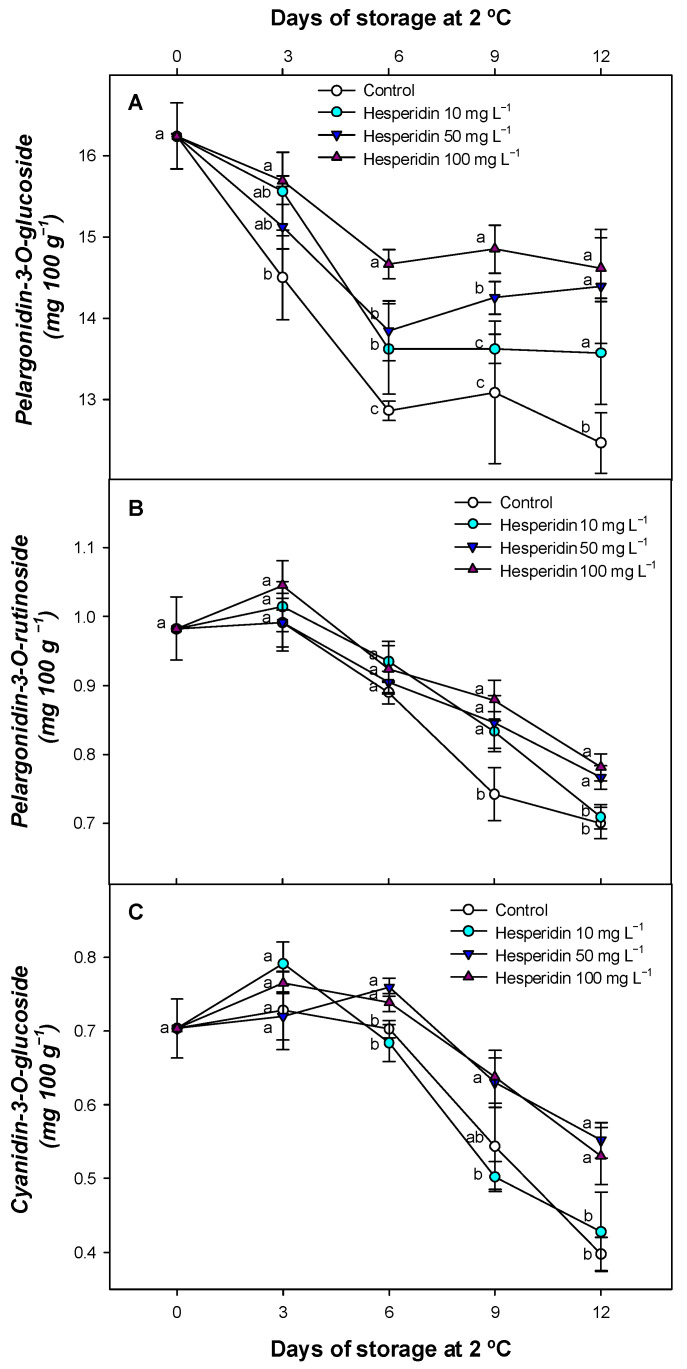
Pelargonidin-3-O-glucoside content (mg 100 g^−1^, (**A**)), pelargonidin-3-O-rutinoside concentration (mg 100 g^−1^) (**B**), and cyanidin-3-O-glucoside (mg 100 g^−1^) (**C**) for untreated strawberries or hesperidin-treated fruit with several concentrations throughout 12 d of cold storage (mean ± SE (*n* = 3)). Lowercase letters, which are different for each sampling period, are significantly different (*p* < 0.05) between postharvest applications.

Among the three anthocyanins analyzed during postharvest storage of strawberries at 2 °C, pelargonidin-3-O-glucoside was the predominant compound, followed by pelargonidin-3-O-rutinoside and cyanidin-3-O-glucoside (Figure 6).

The average contents observed for these anthocyanins were in line with values reported by other authors for strawberries in other studies [60,61,62,63]. Quantitatively, pelargonidin-3-O-glucoside was approximately 15 times more abundant than pelargonidin-3-O-rutinoside, and about 25–30 times more abundant than cyanidin-3-O-glucoside, confirming its important role in strawberry pigmentation. During storage, a general trend of decreasing concentrations was observed for all three anthocyanins. However, a slight increase or stabilization in content was observed for pelargonidin-3-O-rutinoside and cyanidin-3-O-glucoside during the early to mid-storage periods, indicating some resilience under cold conditions. However, treatments with hesperidin significantly delayed the degradation of anthocyanins throughout the storage period, particularly toward the end.

The decrease in anthocyanins in strawberries has been associated with oxidation processes and an increased activity of PPO [64]. In strawberries and red pitahaya, it has been demonstrated that apple polyphenols can prevent the oxidation of anthocyanins in strawberries [16,52]. Other fruit extracts, such as arrayan (*Luma apiculata*) and grape pomace, have been shown to maintain strawberry anthocyanins during storage, with this effect primarily attributed to the antioxidant capacity of the plant extracts [65,66]. For this reason, a delay in hesperidin-treated batches suggests that the antioxidant activity of hesperidin may act as a protective agent, helping to delay the evolution of visual and nutritional properties of strawberries during refrigerated storage.

## 4. Conclusions and Future Perspectives

The present research offers novel evidence supporting, for the first time, the efficacy of hesperidin as a postharvest treatment for preserving strawberry quality under cold storage conditions. Hesperidin applications, even at low concentrations, delayed key physiological and biochemical deterioration processes, including weight loss, softening, membrane oxidation, and pigment degradation, indicating a delay in senescence. In addition, hesperidin treatments effectively modulated respiratory metabolism and ethylene production, both of which are closely linked to quality loss and pigment breakdown. These effects can be attributed to its role in reducing oxidative stress and ethylene biosynthesis, key mechanisms previously associated with senescence control in fruits. Altogether, the results demonstrate that hesperidin, a citrus-derived flavonoid, is a promising and natural postharvest tool for extending strawberry shelf life, maintaining both visual and nutritional quality.

Although recent research has investigated citrus peel extract applications to increase fruit storability, none have specifically addressed the role or quantified the presence of hesperidin, despite being one of the major flavonoids in citrus byproducts such as peels and membranes. This oversight presents an opportunity to explore the contribution of hesperidin itself within these matrices. From a practical and commercial perspective, the application of hesperidin as a postharvest treatment could be particularly attractive if implemented using citrus-derived extracts naturally rich in hesperidin, obtained from agro-industrial byproducts. Applying these extracts at concentrations similar to those tested in this study would provide a cost-effective and eco-friendly strategy, adding value to citrus waste streams while extending the shelf life of strawberries by approximately three days. Following circular economy guidelines, this practice would contribute to the sustainable recovery of resources from food waste and support the development of natural postharvest treatments in the agri-food sector.

## Data Availability

Data are contained within the article. Further inquiries can be directed to the corresponding authors.
